# Schools for improving adolescent health: long way to go

**DOI:** 10.1016/j.lansea.2023.100193

**Published:** 2023-04-05

**Authors:** The Lancet Regional Health – Southeast Asia

Adolescents aged 10–19 years represent 16% of the world's population and 29% of the population in south Asian countries. Their health and wellbeing are key for present and future development. The recent UNESCO report *Ready to learn and thrive* provides a global overview of school health and nutrition (SHN) policies and programmes, showing that these programmes are practical and affordable ways to support children and adolescents during their development period, yet the most disadvantaged children and adolescents who could benefit the most from these programme are missing out.

Currently, more than 20 million adolescents aged 10–14 years in South Asia do not go to school and therefore cannot benefit from SHN programmes. India and Pakistan combined account for more than 80% of south Asia's total children and adolescents who are out of school. This high school absence rate is leading to the highest incidences of child marriage and child labour in the world. UNICEF estimates that 12% (41 million) of the children aged 5–14 years in south Asia are involved in child labour. And significantly, more girls than boys never go to school in south Asia because of disparate gender norms. Rehan and colleagues discuss in their Comment how gender disparity causes absenteeism among Pakistani girls during menstruation.

School dropout exacerbates health risk behaviours in adolescents, which may continue into adulthood, contributing to several of the leading causes of death, disability, and social problems, with health impacts disproportionately higher among girls than boys. As per the fifth National Family Health Survey (2019–21) from India, almost 60% of girls aged 15–19 years in India have anaemia. Almost half of the girls in south Asia are married, one in five give birth before they reach the age of 18 years, and babies born to adolescent mothers are at an increased risk of morbidity and mortality. South Asia is home to the largest number of child brides (28%), with 17% of girls having experienced intimate partner violence. This type of violence also includes violent disciplining, which is considered to be a cultural norm in some parts of the world. In this Article, Aggarwal and colleagues show that poor mental health preceded and was a consequence of child marriage, and call for including mental health in policies and programmes aimed at reducing early marriage. The UNESCO report shows that healthy, well nourished, and happy children and adolescents learn better and are more likely to lead healthy and fulfilling lives. The report also shows that fewer children drop out of school when the learning environments are safe, have proper waste and sanitation facilities, and provide proper meals. Governments have used cash transfers, midday meals, and free transport as incentives to attract children and their parents to school. But there are many children, particularly the most vulnerable and marginalised, who are still missing out. Therefore, there needs to be a strong political will and multisectoral action to encourage these children to attend school through targeted interventions.

With most countries having a policy or programme targeting adolescent health and wellbeing, much of this mortality and morbidity should be preventable. With a large vacuum of health-care support for adolescent health needs, which are different from the needs of children and adults, schools have a huge potential to fill a deep gap in terms of providing information, building awareness, promoting healthy lifestyles, and building life skills for future. In India, the government launched an ambitious Adolescent Reproductive and Sexual Health Strategy (clinic based) in 2005 and the National Adolescent Health Programme (*Rashtriya Kishore Swaasthya Karyakram* or RKSK), which is clinic and community based, in 2014. A rapid review of these programmes in 2016 at the national level and in four states noted that lack of accessibility and awareness about the existence of such programmes were the main barriers to uptake from the demand side. The more recent School Health & Wellness Programme teaches some aspects of health promotion and disease prevention. However, all these programmes fall short of a comprehensive approach for adolescent needs, and there are limited data to show that these programmes have been able to achieve any behavioural outcomes. A study to understand more about the linkages between the school health and nutrition (SHN)programme implementation, impact, and challenges in Nepal unveiled that the programme positively impacted students, schools, and communities; however, their success requires more awareness building and advocacy.

South Asia has the largest adolescent population in the world, comprising 350 million individuals. The safety and wellbeing of children and adolescents must be at the centre of efforts of governments to achieve the Sustainable Development Goals by 2030, and schools play a crucial role in such efforts. Adolescent behaviours are influenced at the individual, peer, family, school, community, and societal levels. Although the UNESCO report cautions against the diffuse action and scattered nature of multisectoral interventions, a collaborative effort that engages multiple partners is necessary to promote the physical and psychosocial health of all young people and to equip them with knowledge and skills to generate human capital for sustainable development. Children and adolescents will reach and stay in school only if nurtured in safe, participative, inclusive, and supportive environments.

